# Hexagonal boron nitride nanomechanical resonators with spatially visualized motion

**DOI:** 10.1038/micronano.2017.38

**Published:** 2017-07-31

**Authors:** Xu-Qian Zheng, Jaesung Lee, Philip X.-L. Feng

**Affiliations:** 1 Department of Electrical Engineering & Computer Science, Case School of Engineering, Case Western Reserve University, 10900 Euclid Avenue, Cleveland, OH 44106, USA

**Keywords:** Hexagonal boron nitride (h-BN), multimode resonances, nanoelectromechanical systems (NEMS), resonators, spatial mapping, Young’s modulus

## Abstract

Atomic layers of hexagonal boron nitride (h-BN) crystal are excellent candidates for structural materials as enabling ultrathin, two-dimensional (2D) nanoelectromechanical systems (NEMS) due to the outstanding mechanical properties and very wide bandgap (5.9 eV) of h-BN. In this work, we report the experimental demonstration of h-BN 2D nanomechanical resonators vibrating at high and very high frequencies (from ~5 to ~70 MHz), and investigations of the elastic properties of h-BN by measuring the multimode resonant behavior of these devices. First, we demonstrate a dry-transferred doubly clamped h-BN membrane with ~6.7 nm thickness, the thinnest h-BN resonator known to date. In addition, we fabricate circular drumhead h-BN resonators with thicknesses ranging from ~9 to 292 nm, from which we measure up to eight resonance modes in the range of ~18 to 35 MHz. Combining measurements and modeling of the rich multimode resonances, we resolve h-BN’s elastic behavior, including the transition from membrane to disk regime, with built-in tension ranging from 0.02 to 2 N m^−1^. The Young’s modulus of h-BN is determined to be *E*_Y_≈392 GPa from the measured resonances. The ultrasensitive measurements further reveal subtle structural characteristics and mechanical properties of the suspended h-BN diaphragms, including anisotropic built-in tension and bulging, thus suggesting guidelines on how these effects can be exploited for engineering multimode resonant functions in 2D NEMS transducers.

## Introduction

Nanoelectromechanical systems (NEMS) vibrating at their resonance modes and made from atomic layer crystalline materials have attracted increasing research interest owing to their promises for exceptionally high responsivities and sensitivities to external stimuli, enabled by their ultralow weight (mass) and ultrahigh surface-area-to-volume ratio^1,2,3^. Following semi-metallic graphene, the early hallmark of two-dimensional (2D) crystals, a variety of 2D materials have been studied as structural materials for 2D NEMS resonators, including superconducting NbSe_2_ (Ref. 4), semiconducting MoS_2_ (Refs. 3,5,6,7), and black phosphorus^8,9^, which opens a wide spectrum of emerging applications, such as sensing^10,11^ and signal processing with ultralow power and broad tunability^12,13^. Although atomic layer crystals with bandgaps ranging from 0 to 2 eV have been studied in earlier explorations (such as 0 eV graphene^1,2,12^, 0.3–1.5 eV black phosphorus^8,9^, 1.2-1.9 eV MoS_2_ (Refs. 3,5,6,7), and so on), 2D NEMS utilizing wide bandgap atomic layer materials have not yet been demonstrated. The adoption of wide bandgap 2D materials in NEMS resonators could offer new opportunities for interactions with ultraviolet (UV) photons and for higher power density-handling capabilities, including higher electrical voltage and higher light intensity.

Ultrathin hexagonal boron nitride (h-BN) crystals isolated from their layered bulk have recently been employed as essential building blocks for emerging 2D devices and heterostructures. The h-BN material has a very wide bandgap (5.9 eV)^14^ and excellent chemical and thermal stability beyond that offered by graphene^15,16^, making h-BN attractive for wide bandgap 2D NEMS resonators. The h-BN crystal also possesses attractive mechanical properties owing to its hexagonal crystal structure nearly identical to that of graphene, including a Young’s modulus theoretically predicted to be as high as *E*_Y_≈780 GPa (Ref. 17), and a very high breaking strain limit of *ε*≈22% (Ref. 18). In addition, monolayer h-BN is theoretically predicted to have strong piezoelectricity, thus showing promise for potential integrated electromechanical actuation and sensing^19,20^. Further, very importantly, the graphene-like in-plane honeycomb crystal structure of h-BN facilitates an ultra-smooth surface and lattice-matched interface with graphene, thus enhancing electron transport in graphene channels to achieve greatly boosted mobility (up to 1,000,000 cm^2^ V^−1^ s^−1^)^21,22,23^. Given these special characteristics, it is natural to choose h-BN as an attractive candidate among 2D crystals for innovating future generations of NEMS and nano-optomechanical systems. To date, h-BN has primarily been employed as lattice-matched high-κ dielectric layers in 2D heterostructures for enabling high-performance electronic devices^21,22,23^, and in UV and deep UV optoelectronic devices^14,24^; however, its excellent mechanical and electromechanical properties have not yet been investigated and remain unexploited in device platforms. This lack of exploitation persists because of technical challenges associated with the fabrication of suspended structures and nanomechanical devices in the h-BN crystal, including greater difficulties in both the isolation of mono- and few-layer flakes (compared with graphene) and the identification of ultrathin h-BN structures (due to its transparency in the visible range)^25^. Equally important, it has also been plagued by the challenges involved in measuring responses from the vanishingly minuscule motion of the free-standing h-BN structures. Therefore, both delicate, deterministic device fabrication and high-precision measurements are greatly desired. Our approach here enables the first fabrication and detection of ultrathin h-BN 2D NEMS resonators, which are the smallest ultrawide bandgap crystalline resonators with demonstrated multimode resonances.

In this work, we develop a precise protocol to efficiently identify and discern very thin pristine h-BN flakes (with thicknesses ranging from ~6.7 to ~292 nm) exfoliated from their layered bulk, and utilize them to fabricate suspended h-BN devices that function as new nanomechanical resonators. We perform comprehensive experimental measurements on the minuscule resonator motion using interferometric detection techniques. By vividly visualizing the static structure and dynamic resonance motion of the device using a high spatial-resolution (⩽1 μm) spectromicroscopy mapping technique with minimized parasitic photothermal effects, we investigate the anisotropic built-in tension and bulging-induced phenomena, such as resonance mode shape symmetry breaking and splitting resonant modes. Moreover, we demonstrate comprehensive determination of the h-BN material’s Young’s modulus from the experimentally measured resonance characteristics.

## Materials and methods

**Figure 1:**
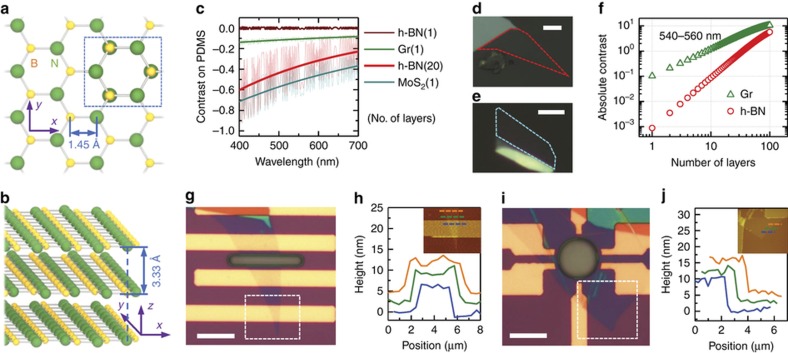
Crystal structure of hexagonal boron nitride (h-BN) and basic characteristics of h-BN nanomechanical resonators. (**a**) Top view of h-BN crystal structure. The box outlined with a blue dashed line illustrates AA′ stacking of the h-BN layered structure. (**b**) Tilted side view of the h-BN crystal structure. (**c**) Optical contrast of two-dimensional (2D) crystals, h-BN, graphene (Gr), and MoS_2_, on a PDMS/glass substrate. The faint lines show the calculated contrast, and the thick solid lines show the exponential fitting as the backbone contrast curve of the corresponding material. (**d**) and (**e**) Optical microscopy images of an h-BN and a MoS_2_ flake on an PDMS/glass substrate with red and cyan dashed lines indicating the 20-layer h-BN and monolayer MoS_2_ region, respectively. The brightness of the images is enhanced by 40%, and the scale bars are 5 μm. (**f**) Dependence on the thickness of the averaged absolute contrast (540–560 nm wavelength range) for graphene and h-BN on a PDMS/glass substrate. (**g**) Optical microscopy image of Device #1, a doubly clamped h-BN resonator suspended over a microtrench 3 μm wide. Scale bar is 10 μm. The h-BN flake before transfer is shown in **d**. (**h**) Atomic force microscopy (AFM) traces of Device #1 corresponding to the dashed lines shown in the inset, that is, the AFM image of the dashed line box area in (**g**). The thickness of the h-BN obtained from the traces is 6.77±0.33 nm. (**i**) and (**j**) Optical image and AFM traces of the circular drumhead h-BN resonator, Device #2, respectively. The thickness of h-BN is 9.84±0.40 nm. Scale bar is 10 μm.

The superior mechanical properties of h-BN are rooted in its graphene-like honeycomb crystal structure. Figures 1a and b illustrate the crystal structure of h-BN, which is formed by replacing carbon atoms in a graphene crystal with boron and nitrogen atoms. Thus, h-BN has nearly the same bond length (1.45 Å) and layer distance (3.33 Å) as graphene does (1.42 Å and 3.35 Å, respectively).

We use a suite of specially developed, completely dry exfoliation, and transfer techniques to fabricate pristine h-BN resonators suspended over pre-defined microtrenches^26^. We obtain h-BN layers a few nanometers thick from high-quality bulk h-BN by exfoliating it onto a polydimethylsiloxane (PDMS) stamp. After exfoliation and careful optical identification, we transfer the h-BN nanosheet, with controlled alignment, to a pre-defined microtrench with the aid of a micromanipulator to achieve a suspended structure. This technique enables the fabrication of pristine suspended h-BN resonators free from wet chemistry contamination compared with conventional wet transfer methods^27,28^. In addition, after all the device fabrication steps, we conduct annealing to further minimize the potential deterioration of device performance due to adsorbates and localized stress.

We analyze the smallest achievable h-BN thickness for the resonator limited by visibility using this device-geometry-controllable transfer technique and, in accordance with the guideline analysis, we fabricated the thinnest possible resonator using this method. The refractive index of h-BN^25,29^ is close to that of a PDMS stamp at 550 nm wavelength^30^. Thus, the reflectance at the interface of the materials is reduced, significantly diminishing the optical contrast of h-BN on the PDMS stamp. In our analysis, monolayer h-BN on PDMS has showed extremely low optical contrast (Figure 1c). In this work, our thinnest h-BN nanosheet identified on PDMS after exfoliation is ~6.7 nm (~20 layers) thick (Figure 1d), using which we have fabricated the thinnest h-BN nanoresonator with exfoliated h-BN crystal, that is, Device #1, a doubly clamped h-BN flake with 6.7 nm thickness across a trench 3 μm wide and 2.2 μm deep. According to theoretical calculations, a 20-layer h-BN flake on PDMS would have an optical contrast comparable to that of monolayer MoS_2_ on PDMS (Figures 1c-e), which approaches the visibility limit for the human eye. Figures 1g and i show representative examples of the fabricated Devices #1 and #2, respectively.

Previously, 2D NEMS resonators were fabricated using semi-metallic graphene^1,2,12^, superconducting NbSe_2_ (Ref. 4), semiconducting MoS_2_ (Refs. 3,5,6,7) and black phosphorus^8,9^. These devices are primarily actuated by electrostatic forces. However, this scheme requires conductive 2D materials to form a capacitor between the freestanding 2D resonator and a back gate and, therefore, cannot be readily applied to insulating h-BN. Similarly, displacement detection via suspended channel transistor^2^ and piezoresistive effects^31^ cannot be used. Meantime, h-BN might offer sufficient piezoelectricity down to a few-layer regime^19,20^, which remains challenging at the device level, whereas the thinnest h-BN device (~20 layers) reported here is not expected to fall in the regime of strong piezoelectricity. Therefore, pure optical detection, and specifically optical interferometry, is a natural scheme for characterization of these first h-BN nanomechanical resonators (Supplementary Information).

Furthermore, although adoption of 2D materials into NEMS is still emerging, many of the intricate structural and elastic properties of such devices are of fundamental interest and are worth exploring as we move toward precise device engineering^6^. Ultrasensitive detection of multimode Brownian motion using high spatial-resolution scanning optical interferometric spectromicroscopy^32^ likely remains the best technique for unveiling these subtle features. To effectively probe the detailed structural properties, less light absorption is always advantageous for minimal parasitic thermal stress induced by photothermal heating, which can obscure the intrinsic device characteristics. Consequently, it is desirable to use wide-bandgap 2D crystals, such as h-BN, for NEMS resonators, in order to observe the higher order Brownian resonances and explore the otherwise hidden structural characteristics of the devices.

## Results

**Figure 2:**
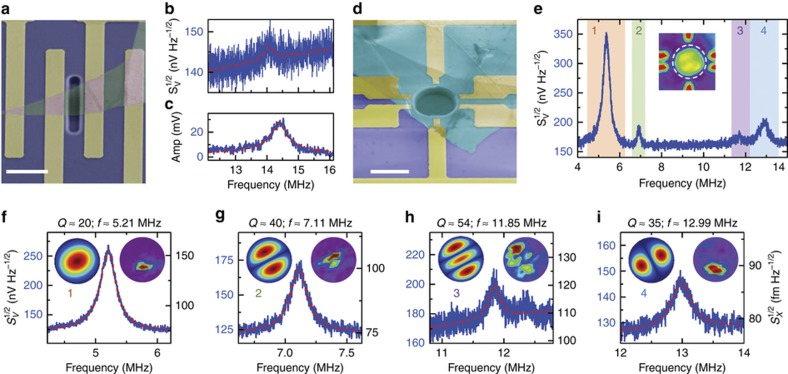
Optical interferometry resonance measurement results for Devices #1 and #2. (**a**) Colored scanning electron microscope (SEM) image of Device #1. (**b**) and (**c**) Measured thermomechanical noise spectrum and photothermally driven resonance spectrum of Device #1, respectively. (**d**) Colored SEM image of Device #2. (**e**) Measured thermomechanical motion spectrum of Device #2. Inset: Static reflectance mapping of device area. (**f**)–(**i**) Zoomed-in spectra of each resonance of Device #2 with insets showing the finite element method (FEM) simulated mode shape (left) and optical interferometric mapping of the spatially resolved device motion for each resonance mode (right). All scale bars are 10 μm.

In the current study, we demonstrate measurements of both undriven thermomechanical resonances that arise from Brownian-motion thermodynamic fluctuations and photothermally driven oscillations of the thinnest Device #1 (Figures 2b and c, respectively) with resonance frequencies of *f*_th_≈14.06 MHz and *f*_drv_≈14.38 MHz and quality factors (*Q*s) of *Q*_th_≈39 and *Q*_drv_≈33, respectively.

Beyond the simple doubly clamped 2D resonator, h-BN resonators with circular drumhead geometry are of greater interest due to their easier availability for multimode resonances. Based on a circular drumhead h-BN resonator (Device #2 shown in Figures 1i and 2d) with a diameter *d*≈11.3 μm and a thickness *t*≈10 nm, we have measured multimode thermomechanical resonances with up to 4 modes and fitted the resonance data to a damped harmonic resonator model using Equation (1):

(1)Sx,th1/2(ω)=(4kBTωmMm,eff⋅Qm⋅1(ω2−ωm2)2+(ωωm/Qm)2)1/2,

where *k*_B_, *T*, *ω*_*m*_, *M*_*m*,eff_, and *Q*_*m*_ are the Boltzmann’s constant, temperature, angular resonance frequency, resonator effective mass, and quality factor of the *m*-th mode, respectively. As shown in Figures 2e–i, the multimode resonance frequencies range from ~5.2 MHz to ~13 MHz with *Q*s from 20 to 54.

To understand the mechanical properties and resonance behaviors of the 2D h-BN resonators in detail, we further investigate the multimode resonances and their vibrational mode shapes using scanning spectromicroscopy techniques^32^. We scan the 633 nm red laser over the device area and measure the amplitudes of reflected light intensity. In the static measurement map (inset in Figure 2e), we find that the reflectance from the resonator shows an uneven pattern over the suspended region. This observation provides important structural information about the device in that, although the suspended h-BN nanosheet appears flat in optical (Figure 1i) and scanning electron microscopy (SEM; Figure 2d) images, mild wrinkles could be present in the diaphragm, as induced by spatially uneven tension. This asymmetric tension might arise from the directional mechanical exfoliation and transfer of h-BN nanosheets during device fabrication (one can define the direction of transfer as the axis perpendicular to the advancing frontline/boundary between the flake’s regions contacted and not-yet-contacted to the substrate that is receiving the flake).

We use spatially resolved mapping of the detailed mode shapes of the measured resonances as a powerful tool for more precise probing and quantification of the rich mechanical properties of these h-BN nanomechanical devices (beyond the basic information of *f* and *Q* values). First, we show that spatial mapping of the mode shapes reveals the asymmetric built-in tension. The right-hand insets of Figures 2f–i represent the spatially resolved motion amplitude at each resonance frequency and clearly show that the mode shapes are more complicated than those expected simply based on device geometry. The antinodes of the 1st, 3rd, and 4th modes deviate from the center of the resonator. In addition, the 3rd mode shape is no longer the degenerated mode of the 2nd mode and consists of two nodal lines in the same direction. We perform finite element method (FEM in COMSOL Multiphysics) simulations to further investigate these unusual resonance mode shapes. Since the device is very thin (~10 nm thick), we assume that the resonances of the device are governed by pre-tension rather than by flexural rigidity. The left insets in Figures 2f–i show the FEM simulation results when we apply asymmetric biaxial tensions (0.29 N m^−1^ and 0.05 N m^−1^ for each direction), and the resonance mode shapes agree with the measured results. Our results clearly demonstrate that, although the asymmetric biaxial built-in tensions in the resonator are ultrasmall (strain levels of ~74 ppm and ~13 ppm), they impact the resonance characteristics and dictate the resonance motion to a great extent.

**Figure 3:**
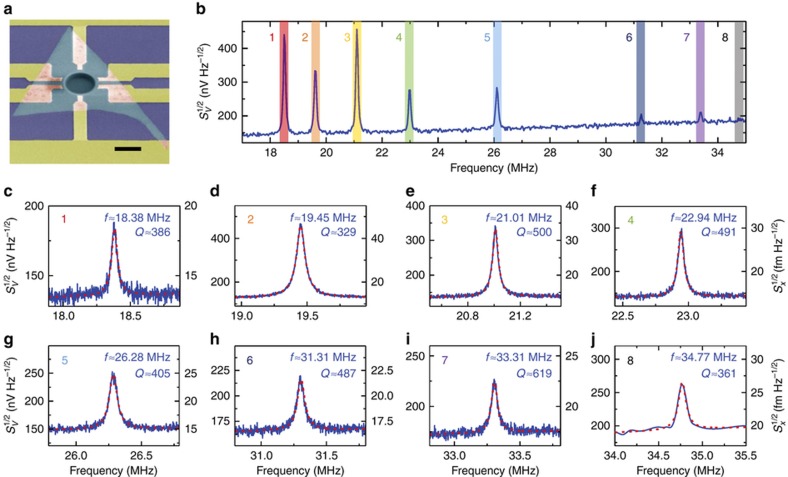
Measured resonance characteristics of Device #3. (**a**) Color-enhanced SEM image of the device. Scale bar is 10 μm. (**b**) Wide-range thermomechanical resonance spectrum with 8 resonance modes. (**c**)–(**j**) Zoomed-in spectra of each thermomechanical resonance mode.

Our further investigation of the mechanical properties of h-BN resonators relative to a thicker drumhead device, that is, Device #3 (*d*≈11.1 μm, *t*≈30 nm), reveals more complicated and intriguing device behavior that is of interest for further device engineering. In our wide-range frequency sweep of the device’s undriven thermomechanical motion, we find multimode resonances up to the 7th mode, with frequencies ranging from ~18.38 to ~33.31 MHz and *Q*s of 329 to 619 (Figure 3). Especially for this resonator, the sensitivity of the measurement system reaches $S_{x,\mathrm{sys}}^{1/2}$≈ 12.9 fm Hz^−1/2^, demonstrating the excellent performance of this system and the associated techniques (Supplementary Information).

**Figure 4:**
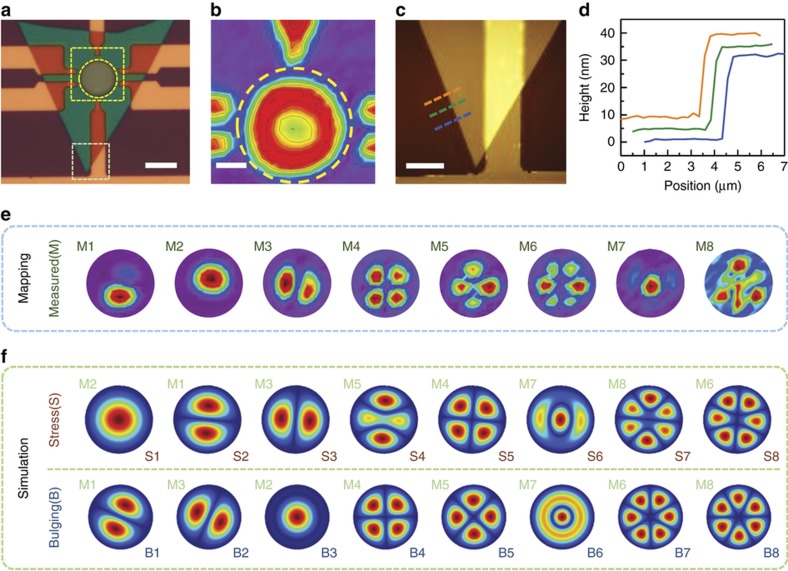
Spatially resolved resonance mode shapes of Device #3. (**a**) Optical microscopy image showing the corresponding areas of (**b**) optical interferometric mapping and (**c**) atomic force microscopy (AFM) scanning within the yellow and light-green outlined boxes, respectively. Scale bars are 10, 3, and 5 μm for **a**–**c**, respectively. (**d**) AFM traces of the device corresponding to the dashed lines in **c** showing an h-BN thickness of 30.46±0.39 nm. (**e**) Optical interferometry mapping of spatially resolved resonator motion for the 8 resonance modes. (**f**) COMSOL Multiphysics simulations for the shapes of different resonance modes with 20 MPa uniaxial pre-stress (1st row) and 143 nm center bulging (2nd row). The numbers indicate the correspondence of simulated mode shapes with measured ones.

We also conduct high spectral- and spatial-resolution scanning spectromicroscopic measurements on Device #3, from which an 8th resonance mode near 35 MHz is found owing to the extensive mapping data. Such high spatial-resolution mapping reveals both structural and motional characteristics of the resonator that have been more difficult to obtain using single- or few-spot interferometric detection. In Figure 4, both static reflectance mapping (Figure 4b) and dynamic displacement mapping of the device thermomechanical motion (Figure 4e) are shown. The structure of the freestanding h-BN is indicated by the static reflectance map, with the lowest reflectance located at the center of the diaphragm and showing a gradual increase towards the clamping edge (Figure 4b). This reflectance gradient implies a non-flat suspended structure of the device, which might arise from 2D material transfer (Supplementary Information). For Device #3, the diaphragm has a thickness of ~30 nm (Figure 4d), which is not sufficiently thick to ignore the built-in tension effects on resonant behavior.

On the basis of the spatial mapping (which has revealed otherwise hidden or unobservable, subtle, and unusual resonance mode shapes and mode sequences (Figure 4e)), we analyze both the built-in tension and structural bulging effects on the device’s resonant behavior using FEM simulations. In Figure 4f, the first row shows the simulated mode shapes of the device with 20 MPa uniaxial pre-stress (equivalent to 0.6 N m^−1^ of surface tension), and the second row illustrates the mode shapes with spherical bulging of the drumhead, where the center deflection is 143 nm. Although the stressed device simulations match better with the measured results in asymmetric mode shapes such as S4 and M5, and S6 and M7, the bulging device simulations show better agreement in the mode shape sequences such as modes B4 and B5 and modes B7 and B8. In addition, we have investigated the frequency ratios of the multimode resonances for the resonator (Supplementary Information). We have found that the measured frequency ratios are much smaller than the theoretical values for flat devices. In other words, the resonance frequencies are closer to each other than expected. Inspired by the static device reflectance implied by the non-flat device structure, we have verified that the decreased mode spacing could be caused by bulging of the diaphragm. The simulations show that the structure with a 143 nm center bulging deflection has the mode spacing that shows good agreement with the measured results (Supplementary Information).

Due to the complexity of the real device structure, it is natural to combine multiple effects at the same time. Thus, resonance characteristics of the device should be affected by both the asymmetric built-in tension and the bulging of the h-BN resonator, which are introduced in the transfer process and are suggested from the reflectance mapping, respectively. The results reveal that these effects should be considered for frequency and mode shape engineering in freestanding 2D crystalline resonators.

**Figure 5:**
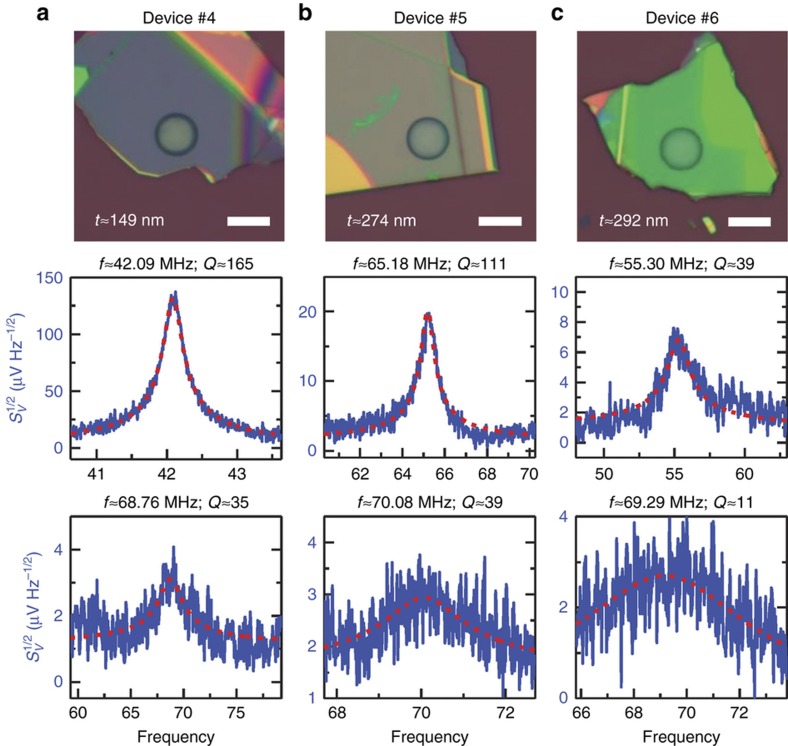
Optical microscopy images and measured photothermally driven resonance spectra of the first 2 modes of (**a**) Device #4, (**b**) Device #5, and (**c**) Device #6. All scale bars are 10 μm.

To further understand the elastic properties of h-BN resonators, we have also fabricated and conducted interferometric measurements on thicker h-BN resonators, that is, Devices #4, #5, and #6 (Figure 5). Due to their larger thickness, the resonance frequencies of these devices are governed by their flexural rigidity (Supplementary Information). Thus, we can estimate the Young’s modulus (*E*_Y_) of the h-BN using the fundamental mode resonance frequencies of these devices. In this type of device, the Young’s modulus can be obtained using Equation (2) (Supplementary Information)^33^:

(2)EY=48π2r4ρ2D(1−v2)[(k0r)2]2⋅t3f02,

where *r* is the radius of the resonator, *ρ*_2D_ is the areal mass density of h-BN, *ν* is the Poisson’s ratio, (*k*_0_*r*)^2^ is an eigenvalue calculated by a numerical method (which in this case is (*k*_0_*r*)^2^=10.215), *t* is the thickness of the device, and *f*_0_ is the fundamental mode resonance frequency.

Since the larger motional masses and higher resonance frequencies of thicker devices make their Brownian motion close to or even smaller than the sensitivity of our measurement system, we photothermally excite these devices to enhance the motion and measure the resonance frequencies, from which 2 resonance modes are detected for each device (Figure 5). Based on the measured fundamental resonances, we have extracted the Young’s moduli of these h-BN devices using Equation (2) and obtained *E*_Y_≈552 GPa for Device #4, *E*_Y_≈377 GPa for Device #5, and *E*_Y_ ≈248 GPa for Device #6. The scattering of the Young’s modulus values could arise from subtle and non-ideal structural effects in these ultrathin and very small drumheads, effects that are not readily and explicitly included in the theoretical model. Nonetheless, we have calculated an averaged Young’s modulus of *E*_Y_=392±125 GPa. This value is lower than the theoretically predicted value^17^ but higher than the results measured in nanoindentation experiments^18^.

## Discussion

**Figure 6:**
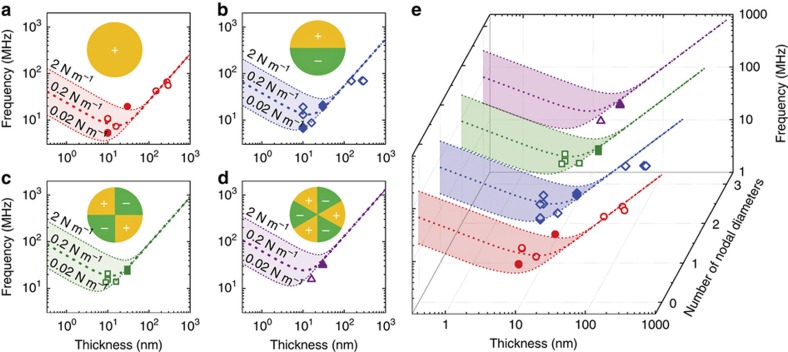
Frequency scaling of circular drumhead hexagonal boron nitride (h-BN) resonators. (**a**–**d**) Resonance frequency and device thickness relations of the fundamental mode and first 3 splitting modes, respectively. Symbols indicate experimental data from optical interferometry measurements. Spatially mapped mode shapes (in experiments) are labeled with solid symbols, and predicted (from resonance sequences) values are indicated by hollow symbols. Insets: Corresponding node distribution illustration of each mode. (**e**) Waterfall plot of the four resonance modes.

With the measured resonance frequencies for circular drumhead devices with thicknesses ranging from 9 to 292 nm, we are able to compare the experimental resonance data and the theoretical frequency scaling. Figure 6 shows the clear elastic transition regimes and frequency scaling of 4 observed modes of the drumhead h-BN resonators using the experimentally determined Young’s modulus of h-BN, that is, *E*_Y_≈392 GPa. In the plots, we use different built-in tensions of 0.02, 0.2, and 2 N/m, which represent the expected range of tension in this type of devices. To calculate the resonance frequency of different modes, we use Equation (3):

(3)fm=(kmr2π)Dρ2Dr4[(kmr)2+γr2D]

where *m* denotes the mode that we calculate, *D* is the flexural rigidity and $D=E_{Y}t^{3}/\left[12\left(1-v^{2}\right)\right]$, and *γ* is the in-plane pre-tension evenly distributed in the 2D material^33^. When the h-BN thickness is less than ~10 nm, where pre-tension dominates, resonance frequencies scale with resonator thickness as *f*∝*t*^-1/2^. When the h-BN thickness is greater than ~100 nm, where the flexural rigidity of the device dominates, the resonance frequencies are proportionally dependent on the resonator thickness, that is, *f*∝*t*. In the transition regime between these regimes, 10 nm<*t*<100 nm, both the pre-tension and flexural rigidity play considerable roles in determining the resonance frequency. In addition, by plotting the experimental resonance frequencies, we find that the experimental results fit the theoretical expectation very well. Thus, for thin devices (*t*<10 nm), we can tune the frequency by using pre-tension engineering, and for thick devices (*t*>100 nm), we can achieve a stable resonator. For devices in the transition regime (10 nm<*t*<100 nm), wide engineering freedom exists for making h-BN nanoresonators.

## Conclusion

In conclusion, we have demonstrated the first h-BN nanomechanical resonators operating at high and very high frequencies with devices covering a wide range of thicknesses (6.7 to 292 nm). Despite the insulating properties, which prohibit electrical detection approaches, we have been able to measure both thermomechanical motion and photothermally driven oscillations of multimode h-BN resonators using the laser-scanning optical interferometry scheme. All devices show robust resonances in the high frequency (HF) or very high frequency (VHF) bands, and we have experimentally determined the Young’s modulus of h-BN, which is *E*_Y_≈392 GPa. Equally importantly, multimode spatial mapping has allowed us to visualize the precise resonance motion of each mode, and these results clearly elucidate otherwise hidden subtle effects, such as uneven built-in tension and bulging in the 2D h-BN diaphragm. This study reveals both important mechanical properties and subtle unusual characteristics of h-BN resonators, adding new understanding and degrees of freedom for engineering of 2D resonators toward advancing applications, such as sensors and multimode signal transduction across mechanical, optical, and electronic domains. This work is expected to pave the way for future investigations into the piezoelectric effects in 2D electromechanical and optoelectromechanical devices made from h-BN and its heterostructures and other piezoelectric 2D crystals.
