# Supplementary material for: Hexagonal boron nitride nanomechanical resonators with spatially visualized motion
Source: Microsyst Nanoeng. 2017 Jul 31;3:17038. doi: 10.1038/micronano.2017.38 (PMC6444998; doi:10.1038/micronano.2017.38)
Supplement: Supplementary Information [file micronano201738-s1.pdf]

Supplementary file

Hexagonal boron nitride nanomechanical resonators with spatially visualized motion

Xu-Qian Zheng, Jaesung Lee and Philip X.-L. Feng

Microsystems & Nanoengineering (2017) **3,** 17038; doi:10.1038/micronano.2017.38; Published online: 31 July 2017

DEVICE FABRICATION

We fabricate hexagonal boron nitride (h-BN) free-standing nanomechanical resonators using a completely dry transfer technique[1]. By utilizing this technique, we are able to fabricate pristine resonators with excellent control and yield, where the two-dimensional (2D) materials undergo no chemical process.

We start the fabrication by preparation of substrate with predefined microstructures. We first deposit 5 nm Cr and 30 nm Au electrodes using photolithography technique onto p++ doped silicon (Si) wafer coated with 290 nm thick thermally grown silicon dioxide (SiO_2). Then, we perform the second step of lithography with dry etching to form 2200 nm deep microtrenches with various geometries. Thus we obtain the substrate with electrode and microtrench patterns ready for 2D material transfer.

We prepare h-BN nanosheets using the mechanical cleavage method. We use Nitto tape for pre-exfoliation iterations and once the exfoliation is sufficient for achieving thin enough hexagonal boron nitride (h-BN) nanosheets (calibrated by bare eyes based on experience), the h-BN flakes are undergoing a final exfoliation onto a polydimethylsiloxane (PDMS) stamp, which is already applied on a glass slide.

Identification of h-BN flakes is then followed. We observe the PDMS stamps with exfoliated h-BN flakes under an optical microscope (Olympus MX50). We choose the h-BN flakes with proper size and thickness, which is evaluated by optical contrast. Based on the flake geometry, we design transfer of a 2D material flake to an ideal substrate microtrench. For example, small flakes could be transferred to small trenches while big flakes to bigger ones; narrow flakes could be ideal for doubly clamped membrane resonators while large uniformly thick nanosheets are better for circular devices.

After the identification, we transfer the h-BN flake onto its designed substrate location with well-engineered orientation using a micromanipulator. We start the transfer process by mounting the target substrate onto the transfer stage with desired orientation and clamping the glass slide onto the micromanipulator. Then, under the optical microscope, we find the identified h-BN flake on PDMS stamp and adjust the micromanipulator to align the flake with targeted substrate patterns through iterations of changing the focus between the h-BN flake and the substrate. The process is followed by bringing down the PDMS stamp toward the substrate using the micromanipulator while the alignment kept being monitored and adjusted. Therefore, the PDMS stamp with h-BN flakes is gradually brought into contact with the substrate. Once the flake is fully in contact, we slowly raise the glass slide as PDMS is peeled away from the substrate while h-BN flake stays due to van der Waals forces. Sometimes, the transfer process is assisted by heating as substrate is heated to around 60 °C to 100 °C during engaging and disengaging the stamp. Thus, the h-BN resonator is fabricated with partially free-standing h-BN nanosheet as designed.

OPTICAL VISIBILITY ON PDMS

To evaluate the visibility of h-BN on the PDMS stamp, we consider the reflectance difference between the bare PDMS stamp and the stamp with the h-BN flake, and calculate the contrast. We investigate interference of incident light in the trilayer structure including h-BN, PDMS, and glass layers (Figure S1). Since the coherence length of halogen lamp in microscope is relatively short compared to the thickness of the glass substrate and the glass substrate is directly placed on the microscope stage during h-BN flake identification, we consider the glass substrate as a pure light absorber, and light reflection at the interface of glass layer and microscope stage has negligible effect upon total light reflected from the structure.

Based on the structure shown in Figure S1, reflectance of the 2D material on stamp can be calculated by[2]

$$R_{2D}(\lambda)=\left|\frac{\boldsymbol{r}_1 e^{i(\phi_1+\phi_2)}+\boldsymbol{r}_2 e^{-i(\phi_1-\phi_2)}+\boldsymbol{r}_3 e^{-i(\phi_1+\phi_2)}+\boldsymbol{r}_1\boldsymbol{r}_2\boldsymbol{r}_3 e^{i(\phi_1-\phi_2)}}{e^{i(\phi_1+\phi_2)}+\boldsymbol{r}_1\boldsymbol{r}_2 e^{-i(\phi_1-\phi_2)}+\boldsymbol{r}_1\boldsymbol{r}_3 e^{-i(\phi_1+\phi_2)}+\boldsymbol{r}_2\boldsymbol{r}_3 e^{i(\phi_1-\phi_2)}}\right|^2, \tag{S1}$$

where, $\boldsymbol{r}_1$, $\boldsymbol{r}_2$, and $\boldsymbol{r}_3$ are reflection coefficients at the air-2D material, 2D material-PDMS, and PDMS-glass interface:

$$\boldsymbol{r}_1(\lambda)=\frac{n_{\text{air}}(\lambda)-\boldsymbol{n}_{2D}(\lambda)}{n_{\text{air}}(\lambda)+\boldsymbol{n}_{2D}(\lambda)},\ \boldsymbol{r}_2(\lambda)=\frac{\boldsymbol{n}_{2D}(\lambda)-n_{\text{PDMS}}(\lambda)}{\boldsymbol{n}_{2D}(\lambda)+n_{\text{PDMS}}(\lambda)},$$
$$r_3(\lambda)=\frac{n_{\text{PDMS}}(\lambda)-n_{\text{glass}}(\lambda)}{n_{\text{PDMS}}(\lambda)+n_{\text{glass}}(\lambda)}, \tag{S2}$$

where n_{air}, $\boldsymbol{n}_{2D}$, n_{PDMS}, and n_{glass} are indices of refraction of air, 2D material, PDMS, and glass, respectively. We used $n_{\text{air}}=1$, $\boldsymbol{n}_{\text{graphene}}=2.6\text{-}1.3i$ (Ref. 2), $n_{\text{PDMS}}(\lambda)=1.417$ (Ref. 3), $n_{\text{glass}}(\lambda)=1.479$ (Ref. 4), $\boldsymbol{n}_{\text{MoS2}}=4.903\text{-}1.063i$ (Ref. 5), and $n_{\text{h-BN}}=2.2$ (Ref. 6) (we use refractive indices at 550 nm, which is in the middle of visible light range for human eyes). For visibility calculation, we use $\phi_1=2\pi n_{2D}d_1/\lambda$, and use $\phi_2=2\pi n_{\text{PDMS}}d_2/\lambda$ for the corresponding phase shifts due to thickness dependent light path differences in each layer. In addition, we use d_1 = 0.335 nm for both monolayer h-BN and graphene, d_1 = 0.70 nm (Ref. 7) for monolayer MoS_2, and d_2 = 0.432 mm for the transparent PDMS layer. The visibility of h-BN flakes on the stamp arises from intensity difference (contrast) between R_{Stamp} and R_{2D} (see Figure S1). R_{Stamp} is the reflectance of bare PDMS with glass which can be calculated using

Department of Electrical Engineering & Computer Science, Case School of Engineering, Case Western Reserve University, 10900 Euclid Avenue, Cleveland, OH 44106, USA.
Correspondence: Philip X.-L. Feng (philip.feng@case.edu)

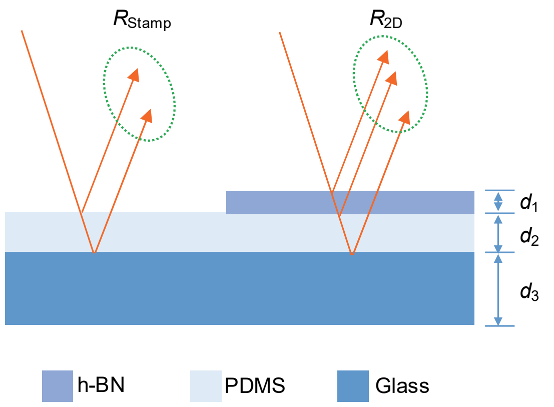

Figure S1 Illustration of reflected light interference for PDMS on glass slide with and without 2D material flake.

Equation (S1) by replacing the refractive index of 2D material $\boldsymbol{n}_{2D}$ with the index of air n_{air}. Thus contrast of exfoliated 2D materials on the stamp is determined by

$$C(\lambda) = \frac{R_{Stamp}(\lambda) - R_{2D}(\lambda)}{R_{Stamp}(\lambda)}. \quad (S3)$$

For quantitative comparison, we calculate optical contrast of monolayer h-BN, 20-layer h-BN, monolayer graphene, and monolayer MoS_2 on PDMS stamp. As shown in Figure 1c in the main text, we plot the contrast of these materials on the stamp in 400 nm to 700 nm wavelength range and extract the backbone of each contrast curve using exponential fitting. The plot shows that while calculated contrast of monolayer graphene on the stamp reaches ~10 % near 550 nm wavelength, the contrast of monolayer h-BN is less than 0.8 % in visible range. We also note that the contrast of 20-layer h-BN is smaller than that of monolayer MoS_2. Based on our experience, we can barely see monolayer MoS_2 flake on PDMS. However, 20-layer h-BN on PDMS stamp has even lower contrast than that of monolayer MoS_2 and it is approaching the limit of human eye visibility through optical microscope.

OPTICAL INTERFEROMETRY

Measurement System

Figure S2 shows the schematic illustration of the customized optical interferometry system used in our experiments. The system consists of a 633 nm He-Ne laser, an amplitude modulated 405 nm blue laser for photothermal driving, a set of lenses, filters and beam splitters to set up light paths, a photo detector (PD) with a spectrum analyzer and a network analyzer to detect resonances of the devices, a customized vacuum chamber with vacuum gauge and optical window, and a high-precision motorized stage for areal scanning of devices. All the measurements in this work are conducted in a moderate vacuum of ~40 mTorr.

The interfaces of materials act as micro beam splitters and mirrors. Thus the light interference occurs when light travels to our devices and total reflectance depends on phase differences created by the distance of gap between h-BN and the microtrench bottom surface. When the suspended h-BN is vibrating at resonance and the bottom surface of the microtrench is steady, thickness of the gap is

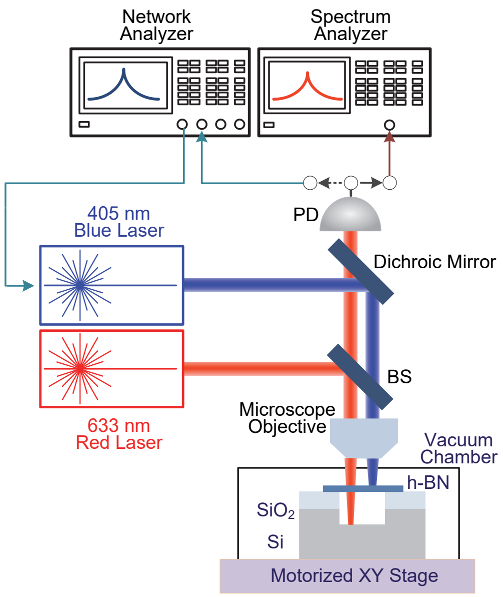

Figure S2 Schematic of the optical interferometry system.

periodically changing due to the periodic mechanical displacement of h-BN, generating phase variation in the interferometry. This phase variation creates intensity change of the overall reflected light. By detecting the reflected light using the PD, we can measure the voltage signal transduced from the mechanical displacement of the resonator. The spectrum analyzer is used to read out the voltage spectra of this signal from PD and further analysis is done on the spectra to acquire the displacement-domain Brownian motion of the h-BN drumhead resonators.

Furthermore, we conduct ultrasensitive motion detection via scanning spectromicroscopy with high spectral and spatial resolutions. The method is realized by scanning the device area with 633 nm laser and measuring the reflected light spectra for arrays of points within the area. By post processing for spectral density of each resonance mode, we are able to map out the relative displacement amplitude of each resonance mode for the device. Mapping of these thermomechanical vibrations vividly visualizes the shapes and textures of high-order Brownian motion in the h-BN resonators. The mapping results of Device #2 and Device #3 are shown in Figure 2 and Figure 4 of the main text, respectively.

Photothermal Driving

In addition to undriven thermomechanical noise detection, the device can be photothermally driven by shining the amplitude modulated 405 nm blue laser on the device area, and resonance motion is read out using the 633 nm red laser. A network analyzer is used in this measurement scheme for modulation of the 405 nm laser and plotting the reflected red laser light spectrum (Figure S2).

Figure S3k-n show the photothermally driven resonance data measured from Device #3 with different driving strength from 20 mV to 200 mV. Amplitude of driven resonances is linearly increased with respect to driving strength applied from the network analyzer; and we do not observe clear nonlinearity. We calculate measured linear operation ranges of multimode resonances by comparing amplitude

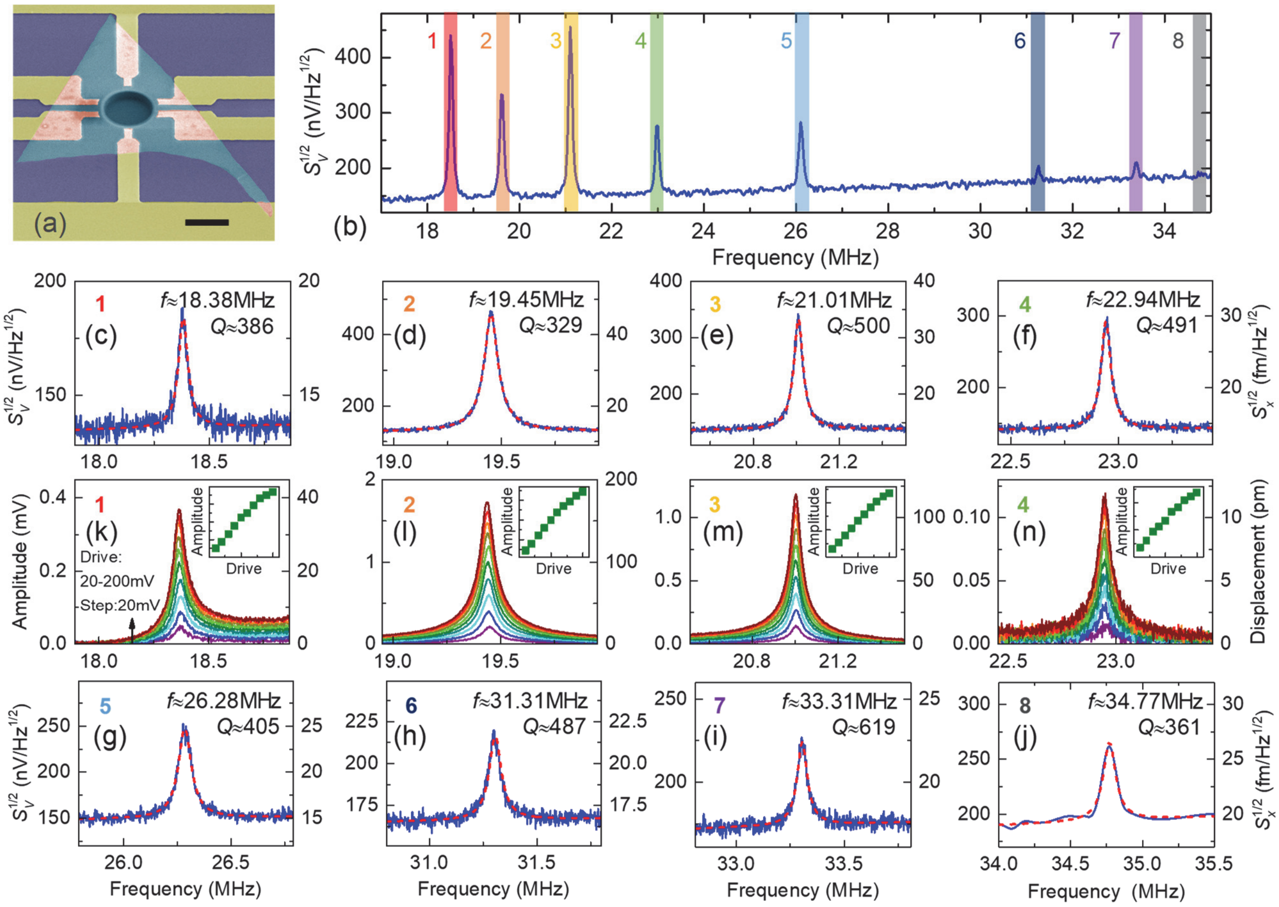

Figure S3 Resonance characteristics of Device #3. (**a**) False colored SEM image of the device. Scale bar is 10μm. (**b**) Wide range undriven thermomechanical resonance spectrum with 8 resonance modes. (**c**-**j**) Zoomed-in spectra of each thermomechanical resonance mode. (**k**-**n**) Photothermally driven resonance spectra measured from the first 4 modes, respectively, each with an inset showing its peak amplitude dependence on drive.

of thermomechanical noise and resonances at the highest driving strength (200 mV). The calculated linear operation ranges are ~67 dB, ~72 dB, ~71 dB, and ~52 dB for 1st, 2nd, 3rd, and 4th mode, respectively. Since no clear Duffing nonlinearity is observed, actual dynamic ranges, linear regimes from thermomechanical noise to onset of nonlinearity, could be much larger than measured linear response ranges.

Responsivity Analysis

We conduct a careful calculation on reflectance of the device to estimate responsivity from mechanical displacement to light reflectance. Similar to the analysis in the former section of supplementary information, we use Equation (S1) and employ r_1, r_2 and $\boldsymbol{r}_3$ as reflection coefficients at the vacuum-h-BN, h-BN-vacuum, and vacuum-silicon interfaces, respectively:

$$r_1(\lambda)=\frac{n_{\mathrm{vac}}(\lambda)-n_{\mathrm{h\text{-}BN}}(\lambda)}{n_{\mathrm{vac}}(\lambda)+n_{\mathrm{h\text{-}BN}}(\lambda)},\; r_2(\lambda)=\frac{n_{\mathrm{h\text{-}BN}}(\lambda)-n_{\mathrm{vac}}(\lambda)}{n_{\mathrm{h\text{-}BN}}(\lambda)+n_{\mathrm{vac}}(\lambda)},$$
$$\boldsymbol{r}_3(\lambda)=\frac{n_{\mathrm{vac}}(\lambda)-\boldsymbol{n}_{\mathrm{Si}}(\lambda)}{n_{\mathrm{vac}}(\lambda)+\boldsymbol{n}_{\mathrm{Si}}(\lambda)}, \tag{S4}$$

and ϕ_1, ϕ_2 are the corresponding phase shifts:

$$\phi_1 = 2\pi n_{2D} t/\lambda,\;\; \phi_2 = 2\pi n_{\mathrm{vac}} d_{\mathrm{vac}}/\lambda. \tag{S5}$$

In the above equations, t is the h-BN thickness, d_{vac} is the vacuum gap depth, λ is the laser wavelength, and n_{vac}, $n_{\mathrm{h\text{-}BN}}$ and $\boldsymbol{n}_{\mathrm{Si}}$ are the refractive indices of vacuum, h-BN and silicon, respectively.

Using the equations, we calculate the reflectance *R*'s dependence on vacuum gap d_{vac} (Figure S4a-b). We use parameters of $\lambda = 633$ nm, $t = 30$ nm, $d_{\mathrm{vac}} = 2200$ nm, $n_{\mathrm{vac}} = 1$, $n_{\mathrm{h\text{-}BN}} = 2.2$ and $\boldsymbol{n}_{\mathrm{Si}} = 3.881\text{-}0.019i$. We also extract the "displacement-to-optical-reflectance" responsivity, which is around -0.741 %/nm at 2200 nm in Figure S4b. In addition, we plot the responsivity dependence on vacuum gap d_{vac}, and find the best responsivity of -0.846 %/nm when d_{vac} is 2218 nm (Figure S4c).

We analyze measured Brownian motion of devices to calibrate the overall responsivity of interferometry system from mechanical displacement to electrical signal. According to the mode shape maps shown in Figure 4e in the main text, the 2nd mode has the mode shape of the fundamental mode; and we use this mode to analyze the responsivity and sensitivity of the resonator.

From Figure 3d in main text, noise level of the measurement system

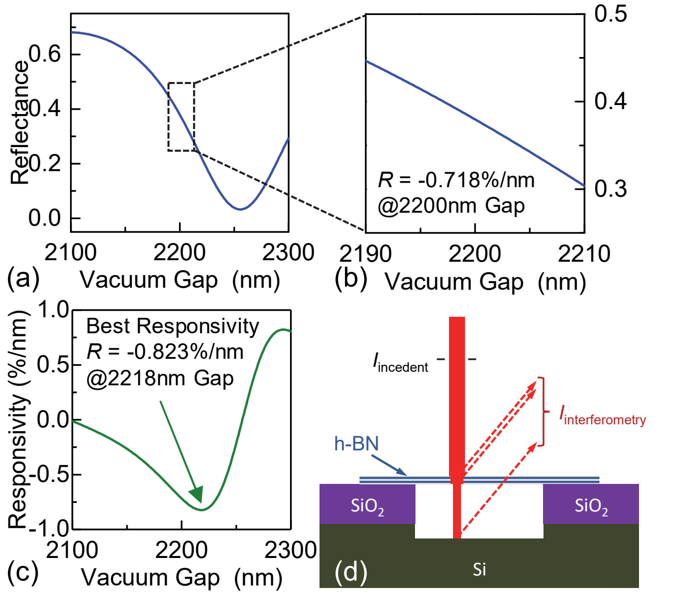

Figure S4 Reflectance of red (633 nm) laser on device with vacuum gap dependency (based on Device #3). (**a**) & (**b**) The relation of reflectance and vacuum gap in 2100-2300 nm and 2190-2210 nm range, respectively. (**c**) Responsivity of reflectance (derivative of reflectance) with air gap in 2100-2300 nm range. (**d**) Schematic illustration of incident light interference in the h-BN resonator device configuration.

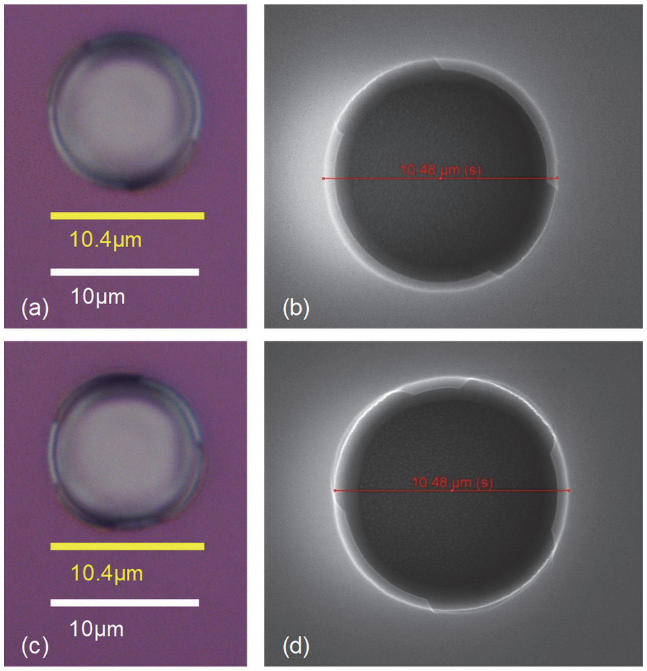

Figure S5 Calibration of the diameters of the circular microtrenches. (**a**,**c**) Optical microscope images with corresponding optically calibrated microtrench diameters (yellow bars and text) and scale bars (white bars and text). (**b**,**d**) SEM images with measured microtrench diameters corresponding to the images on the left panels, respectively.

is $S_{v,\mathrm{sys}}^{1/2} = 134.4\ \mathrm{nV/Hz^{1/2}}$ and measured total voltage spectral density at resonance is $S_{v,\mathrm{total}}^{1/2} = 466.6\ \mathrm{nV/Hz^{1/2}}$. Thus thermomechanical motion in voltage domain is $S_{v,\mathrm{th}}^{1/2} = \left(S_{v,\mathrm{total}} - S_{v,\mathrm{sys}}\right)^{1/2} = 446.8\ \mathrm{nV/Hz^{1/2}}$.

We calculate the thermomechanical noise spectrum in the displacement domain using

$$S_{x,\mathrm{th}}^{1/2}(\omega) = \left(\frac{4k_B T \omega_m}{M_{m,\mathrm{eff}} \cdot Q_m} \cdot \frac{1}{\left(\omega^2 - \omega_m^2\right)^2 + \left(\omega\omega_m / Q_m\right)^2} \right)^{1/2}. \tag{S6}$$

When the device is on resonance ($\omega = \omega_m$), the expression simplifies to

$$S_{x,\mathrm{th}}^{1/2}(\omega_m) = \left(\frac{4k_B T Q_m}{\omega_m{}^3 M_{m,\mathrm{eff}}} \right)^{1/2}, \tag{S7}$$

where ω_m, k_B, T, Q_m, $M_{m,\mathrm{eff}}$ are angular resonance frequency, Boltzmann's constant, temperature, quality factor, and effective mass, respectively, and m denotes the mth mode of the resonator.

We assume laser heating is minimal due to wide bandgap of h-BN and temperature remains at room temperature ($T \approx 300$ K). We also assume that Device #3 is in disk regime, then for the circular clamped disk resonator, the effective mass of fundamental mode resonance is $M_{\mathrm{eff}} = 0.1828M$, where M is the still mass of the resonator. From the Equation (S7), we get $S_{x,\mathrm{th}}^{1/2}(f_0) = 42.6\ \mathrm{fm/Hz^{1/2}}$ for the fundamental mode (2nd lowest mode of Device #3 shown in Figure 2e). By using aforementioned analysis, we determine the responsivity of the optical interferometry system $\Re \equiv S_{v,\mathrm{th}}^{1/2} / S_{x,\mathrm{th}}^{1/2} = 10.5\ \mathrm{nV/fm}$ and system sensitivity $S_{x,\mathrm{sys}}^{1/2} = S_{v,\mathrm{sys}}^{1/2} / \Re = 12.9\ \mathrm{fm/Hz^{1/2}}$. Since the responsivity of the system is the same for all modes (if we assume this is true in certain frequency range), the displacement of each mode can be calculated.

DETERMINATION OF DRUMHEAD RESONATOR DIAMETER

To accurately determine the diameters of the resonators, we conduct multiple measurements using different tools, including optical microscope and SEM, to calibrate the effective diameters of the fabricated circular microtrenches for suspended h-BN resonators. In theory, SEM can achieve ~10 nm and even better resolution, while the resolution of the 100X optical microscope is around 200 nm. Figure S5 shows our calibration results using both optical microscope and SEM on different circular microtrenches for their outer diameters. The results show an <200 nm error from optical microscope measurement compared to SEM results, which is consistent with the theoretical expectation according to the resolution of the optical microscope. For devices in this work (~10 μm in diameter), <200 nm imprecision corresponds to an error below 2 %, which is sufficient for device physics calibration.

DEVICE ELASTIC PROPERTY ANALYSIS AND FREQUENCY SCALING

We analytically calculate the resonance frequency scaling of circular drumhead h-BN resonators. The resonance frequency of a circularly

clamped 2D resonator is

$$f_m = \left(\frac{k_m r}{2\pi}\right)\sqrt{\frac{D}{\rho_{2D} r^4}\left[\left(k_m r\right)^2 + \frac{\gamma r^2}{D}\right]}, \tag{S8}$$

where m denotes the mode, $(k_m r)^2$ is the eigenvalue which can be numerically calculated, r is the radius of circular resonator, D is the flexural rigidity and $D = E_Y t^3 / \left[12\left(1-\nu^2\right)\right]$, ρ_{2D} is the areal density of h-BN flake, and γ is the pre-tension evenly distributed in the 2D material plane[8]. The eigenvalue $(k_m r)^2$ can be determined using the equation

$$\left(k_m r\right)^2 = \alpha + \left(\beta - \alpha\right) e^{-\eta \exp\{\delta \ln(x)\}}, \tag{S9}$$

where $x = \gamma r^2/D$, and for different modes, the eigenvalue is calculated using numerically computed parameters α, β, η and δ given in Ref. 8. In addition, if the device is ideal membrane or ideal disk, this eigenvalue equals to α or β, respectively.

In the regime that flexural rigidity governs, the large flexural rigidity D makes the $\gamma r^2/D$ term in Equation S8 to be minimal and Equation (S8) becomes

$$f_m = \frac{\left(k_m r\right)^2}{2\pi r^2}\sqrt{\frac{D}{\rho_{2D}}}. \tag{S10}$$

Then Young's modulus can be determined by

$$E_Y = \frac{48\pi^2 r^4 \rho_{2D}\left(1-\nu^2\right)}{\left[\left(k_0 r\right)^2\right]^2 \cdot t^3} f_0^2, \tag{S11}$$

from the fundamental mode frequency. Here, $(k_0 r)^2 = 10.215$ for the fundamental mode of disk resonator. Using Equation (S11), we derive Young's modulus of h-BN from the measured resonances. Here, we use $\rho_{2D} = 6.3$ μg/cm^2 = 63 fg/μm^2 as the areal mass density, and $\nu = 0.211$ as Poisson's ratio[9]. Device #4 has a diameter of 10.5 μm and a thickness of 149 nm with a fundamental mode resonance frequency

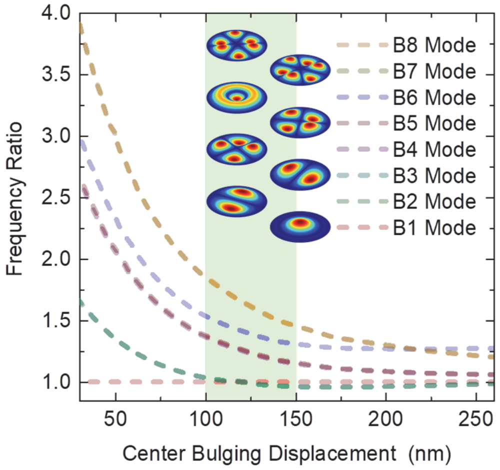

Figure S6 Effect of bulging on resonance frequencies, in terms of frequency ratio normalized by the fundamental mode resonance frequency. The regime with expected frequency ratio is marked light green and the legend shows the corresponding mode shape of each resonance.

Table S1 List of h-BN resonator devices and their parameters

Device #	Diameter d (μm)	Thickness t (nm)	Mode #	Resonance Frequency f_0 (MHz)	Quality Factor Q	Notes
1	3 (Length)	6.8	1	14.06	39	
			1*	14.38	33	*Driven
2	11.3	9.8	1	5.21	20	After AFM Imaging of Suspended Area
			2	7.11	44	
			3	11.85	54	
			4	12.99	35	
2'			1	5.37	25	Before AFM Imaging of Suspended Area
			2	6.59	29	
			3	13.54	41	
3	11.1	30	1	18.38	386	
			2	19.45	329	
			3	21.01	500	
			4	22.94	491	
			5	26.28	405	
			6	31.31	487	
			7	33.31	619	
4	10.5	149	1*	42.09	163	*Driven
			2*	68.76	35	
5	10.4	274	1*	65.18	111	*Driven
			2*	70.08	39	
6	10.5	292	1*	55.30	39	*Driven
			2*	69.29	11	
7	9.8	10	1	10.74	51	After AFM Imaging of Suspended Area
			2	13.19	63	
			3	15.38	77	
7'			1	10.38	99	Before AFM Imaging of Suspended Area
			2	19.07	98	
			3	20.85	68	
8	10.2	16	1	7.31	99	
			2	8.76	81	
			3	13.97	89	
			4	15.81	68	
			5	24.88	130	

Note: The measurement results are from undriven thermomechanical resonances, unless otherwise stated.

of 42.09 MHz, so the calculated Young's modulus of h-BN is E_Y = 552Gpa (Figure 5). For Devices #5 and #6, they have 10.4 μm and 10.5 μm diameters and thicknesses of 274 nm and 292 nm, with measured resonance frequencies 65.18 MHz and 55.30 MHz, and estimated Young's moduli 377 GPa and 248 GPa, respectively (Figure 5). From Equation (S11), the diameter (radius) of resonator is very critical for Young's modulus extraction due to its 4th power scaling. Therefore, we calibrate the diameters carefully using the optical method in the former section. In addition, to minimize the possible interference from adsorbates on the device (which may affect the mass density in Equation (S11)), we conduct device annealing after all the device fabrication. In summary, the averaged Young's modulus achieved from these three devices is 392±125 GPa, which is lower than theoretically predicted values[10] but higher than the results measured by using the nanoindentation method[11].

In the regime where built-in tension dominates, the term $\gamma r^2/D >> (k_m r)^2$, thus Equation (S8) becomes

$$f_m = \frac{(k_m r)}{2\pi r}\sqrt{\frac{\gamma}{\rho_{2D}}}. \quad \text{(S12)}$$

We find that in the membrane regime, the resonance frequency is following power law of $f \propto t^{-1/2}$, while in the disk regime, the resonance frequency is proportional to the device thickness, $f \propto t$.

We analyze the dependency of resonance frequencies in different modes on device thickness for h-BN circular drumhead resonators by plotting f versus t function using Equation (S8) (Figure 6 in main text). We use E_Y = 392 GPa, as extracted from experiments and radius r = 5 μm since all the circular devices have the similar diameter. The plots show clear membrane regime when device thickness is smaller than ~10 nm, disk regime when device thickness is larger than ~100 nm, and a transition regime in between. By plotting the experimental data along with the theoretical frequency scaling, the experiment results show good agreement with the theoretical expectation.

EFFECT OF BULGING ON FREQUENCY SPACING

In Figure 4b of the main text, we find that the reflectance in the center of the circular device is lower than that of the outer part. Since the bottom of the microtrench is flat, we can expect the device to be non-flat according to the reflectance to gap relation showed in Figure S4a. We investigate the effect of bulging on multimode frequency spacing using finite element method (FEM) simulations. Among our h-BN devices we employ Device #3 described in Figures 3 & 4 since we are able to measure the highest vibration harmonics up to the 8th mode in this device. First we analytically calculate mode spacing of an edge clamped circular resonator without bulging. In the membrane model, resonance frequency can be calculated using Equation (S12). Then, the frequency ratio of the fundamental mode and the first 3 splitting modes in the membrane model is $f_0 : f_1 : f_2 : f_3$ = 1 : 1.59 : 2.14 : 2.65, where the subscript index represents the number of nodal diameters of each resonance mode. Similarly, for resonance frequency of edge clamped circular disk, we can use Equation (S10) to calculate the frequency ratio, which is $f_0 : f_1 : f_2 : f_3$ = 1 : 2.08 : 3.41 : 5.00. From our measurement results, however, multimode resonance frequencies are very close to each other and their frequency ratio is $f_1 : f_0 : f_{1'} : f_2 : f_{2'} : f_3 : f_{3'}$ = 0.94 : 1 : 1.08 : 1.18 : 1.35 : 1.61 : 1.79; and its frequency spacing is smaller than the frequency spacing from analytical calculations.

To further understand the reduced frequency spacing in the experimental results, we study frequency ratio of h-BN resonators using FEM simulation. We use the disk model in simulation because Device #3 operates close to the disk regime (see main text Figure 6). On top of that, we model device structure as a spherical cap due to bulging. Figure S6 shows calculated frequency ratio using FEM simulation. It clearly shows that the resonance frequency ratio significantly depends on the bulging deflection; and it decreases as the bulging deflection increases. By simulating the mode shapes of Device #3 with different bulging levels, we find that when the bulging deflection is 143 nm at the circle center, the mode shapes match the mapping results (Figure S6) the best, with frequency spacing of $f_1 : f_{1'} : f_0 : f_2 : f_{2'} : f_3 : f_{3'}$ = 0.97 : 0.97 : 1 : 1.17 : 1.18 : 1.50 : 1.50, which is also very close to the measurement results.

DEVICE SUMMARY

Here we summarize the parameters and performance of all the devices in this paper in Table S1. All the resonance frequencies listed in the table are plotted in Figure 6 of the main text.

For Device #2 and Device #7, we conduct the measurements both before and after AFM imaging of the suspended areas, since the imaging may change the built-in tension of the devices. For the rest of devices, we manage to avoid the built-in tension change induced by the AFM tip by conducting the AFM imaging in the areas of h-BN that are not suspended. Also, we conduct photothermal driving measurements on devices either for better resonance spectrum (Device #1), or because of the ultra-small displacement of the device on resonance (Device #4, Device #5 and Device #6).

REFERENCES

1 Yang R, Zheng X-Q, Wang Z, Miller C J, Feng P X-L. J. Vac. Sci. Technol. B 2014; **32**: 061203.
2 Blake P, Hill E W, Castro Neto A H, Novoselov K S, Jiang D, Yang R, Booth T J, Geim A K. Appl. Phys. Lett. 2007; **91**: 063124.
3 Qiu W. Master Thesis, Louisiana State University, Louisiana 2012.
4 Gao L, Lemarchand F, Lequime M. J. Europ. Opt. Soc. Rap. Public. 2013; **8**: 13010.
5 Beal A R, Hughes H P. J. Phys. C: Solid State Phys. 1979; **12**: 881.
6 Gorbachev R V, Riaz I, Nair R R, Jalil R, Britnell L, Belle B D, Hill E W, Novoselov K S, Watanabe K, Taniguchi T, Geim A K, Blake P. Small 2011; **7**: 465.
7 Splendiani A, Sun L, Zhang Y, Li T, Kim J, Chim C-Y, Galli G, Wang F. Nano Lett. 2010; **10**: 1271.
8 Suzuki H, Yamaguchi N, Izumi H. Acoust. Sci. Technol. 2009; **30**: 348.
9 Boldrin L, Scarpa F, Chowdhury R, Adhikari S. Nanotechnology 2011; **22**: 505702.
10 Pakdel A, Bando Y, Golberg D. Chem. Soc. Rev. 2014; **43**: 934.
11 Song L, Ci L, Lu H, Sorokin P B, Jin C, Ni J, Kvashnin A G, Kvashnin D G, Lou J, Yakobson B I, Ajayan P M. Nano Lett. 2010; **10**: 3209.
